# Biliothoracic Fistula after Microwave Ablation of Liver Metastasis : Literature Review

**DOI:** 10.1155/2021/9913076

**Published:** 2021-05-11

**Authors:** Valentina Tassi, Claudia Mosillo, Massimiliano Mutignani, Roberto Cirocchi, Mark Ragusa, Sergio Bracarda, Giovanni Passalacqua, Gabriele Marinozzi, Massimiliano Allegritti

**Affiliations:** ^1^Thoracic Surgery Unit, Azienda Ospedaliera Santa Maria, Terni, Italy; ^2^Medical and TranslationalOncology Unit, Azienda Ospedaliera Santa Maria, Terni, Italy; ^3^Digestive Endoscopic Unit, Ospedale Niguarda, Milano, Italy; ^4^Department of Medicine and Surgery, University of Perugia, Perugia, Italy; ^5^Division of Interventional Radiology, Azienda Ospedaliera Santa Maria, Terni, Italy; ^6^Digestive Endoscopic Unit, Azienda Ospedaliera Santa Maria, Terni, Italy

## Abstract

Microwave ablation is a safe and effective interventional approach, widely used in the treatment of unresectable primary or metastatic hepatic lesions. Thoracobiliary fistula is a rare postablation complication that can be treated with a conservative or surgical approach. We reviewed aetiology, pathogenesis, clinical picture, diagnostic possibilities, and therapeutic options for biliothoracic fistula developed after microwave ablation of liver metastasis. Furthermore, we reported our experience of successful conservative management of a nonhealing thoracobiliary fistula occurred after percutaneous thermal ablation of colorectal cancer liver metastasis. Our case supports a conservative approach based on percutaneous biliary system decompression and synthetic glue embolization for the treatment of combined biliopleural and biliobronchial fistula.

## 1. Biliothoracic Fistula

Almost 30% of patients with metastatic colorectal cancer (CRC) present with liver metastases only. Although surgical resection is the most effective treatment, patients with isolated CRC liver lesions, deemed to be unresectable, may benefit from thermal ablation (Radiofrequency Ablation, RA, and Microwave Ablation, MWA) or Trans-Arterial Chemoembolization (TACE) [1–3].

Thoracobiliary fistula (TBF) is a rare postablation complication characterized by relevant mortality and morbidity rates [4, 5]. TBF may communicate with either the pleural space (pleurobiliary fistula, PBF) or the bronchi (bronchobiliary fistula, BBF) [6]. Treatment options include surgery, with thoracoabdominal exploration, and conservative therapy characterized by percutaneous transhepatic (PT) or endoscopic retrograde cholangiopancreatography (ERCP) biliary drainage [6–8]. To date, guidelines for the optimal approach of TBF are lacking [7].

We report our experience on combined BPF and BBF treated conservatively in a patient with CRC single liver metastasis undergone MWA.

## 2. Our Experience

**Figure 1:**
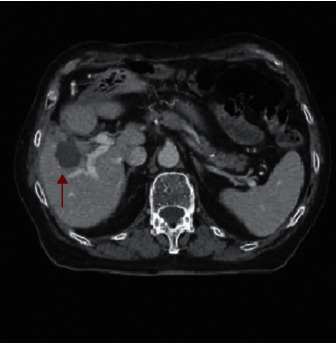
CT imaging of the liver lesion in segment V measuring 39 mm (arrow) that was treated with MWA.

In January 2020, a 73-year-old man with a history of metachronous and unresectable CRC liver metastasis (Figure 1) was admitted to our center because of fatigue and shortness of breath. Since March 2016, the patient has been treated with a multidisciplinary approach characterized by chemotherapy plus targeted therapy and MWA. In particular, MWA of the single hepatic lesion at segment V was performed twice: in October 2016 and November 2019.

**Figure 2:**
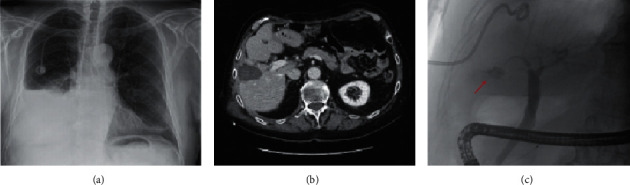
Chest plain radiograph 2 months after MWA showing a right pleural effusion (a); CT scan revealing a hypodense oval lesion of 48 × 33 mm with hyperdense core consistent with postablation liver damage (b); and ERCP demonstrating contrast leakage from right-sided bile duct (arrow) and extravasation of contrast into the right pleural cavity (C).

**Figure 3:**
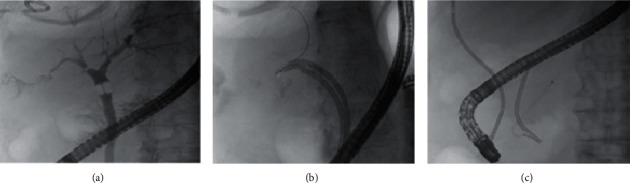
Endoscopic treatment of the BPF by means of a large sphincterotomy (a), placement of two biliary stents (AdvanixBiliary stent, Boston Scientific® 10 Fr x 12 cm) to decompress the right bile duct (b), and a bilioduodenal endoprosthesis (c).

**Figure 4:**
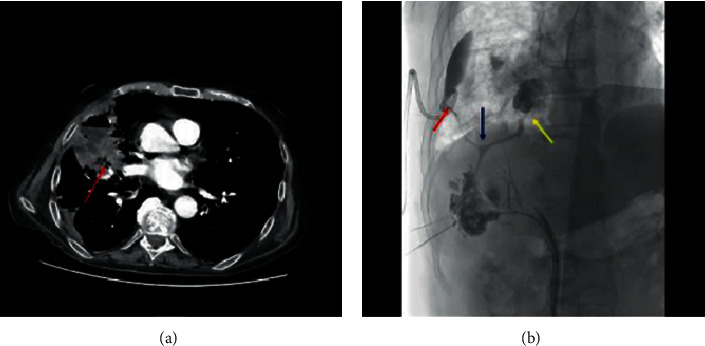
Chest-abdomen CT scan showing a 50 mm-diameter abscess of the middle lobe communicating with the subsegmental bronchi (arrow) (a); percutaneous cholangiography revealing a “Y”-shaped fistula originating from intrahepatic bilioma (blue arrow) and divided in two arms which reached separately the pleural space (red arrow) and the middle lobe abscess up to one segmental bronchus (yellow arrow) (b).

**Figure 5:**
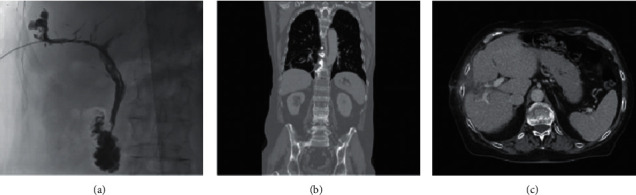
Percutaneous cholangiography (a) and chest-abdomen CT scan (b, c) showing resolution of previous bile leakage.

At the time of admission, the chest X-rays showed right-side pleural effusion (Figure 2(a)) and the thoracentesis revealed bilious fluid; thus, a chest drainage was inserted. A chest and abdomen CT scan confirmed the pleural effusion and revealed postablation liver damage (Figure 2(b)). The clinical hypothesis of BPF was demonstrated by means of ERCP with contrast extravasation from the liver towards the pleural cavity (Figure 2(c)). After sphincterotomy, a bilioduodenal endoprosthesis and multiple biliary stents (AdvanixBiliary stent-Boston Scientific® 10 Fr x 12 cm) were placed to decompress the intrahepatic biliary system adjacent the ablated lesion (Figure 3(a)–3(c)). After this procedure, the amount of biliary fluid from the chest tube progressively decreased and chest X-rays demonstrated the reduction of pleural effusion; thus, the chest tube was removed after two weeks, and the patient was discharged home. Ten days later, he was readmitted at our hospital with fever, dyspnea, and productive cough with greenish sputum. Blood examination showed elevated value of white blood cells count and C-reactive protein. Bacteria culture of blood revealed *Enterococcus faecium*. The chest and abdomen CT scan showed a large lung abscess of the middle lobe communicating with a bilioma of the hepatic segments V–VIII by means of a long and thin BBF (Figure 4(a)). The aforementioned findings were confirmed by the percutaneous cholangiography. This exam revealed a “Y”-shaped fistula originating from the intrahepatic bilioma and dividing in two arms which reached separately the pleural space (BPF) and the middle lobe abscess up to one segmental bronchus (BBF). Moreover, bilioduodenal endoprosthesis obstruction was observed (Figure 4(b)). Immediately, the pleural space and the middle lobe abscess were drained by two chest tubes (10Fr pigtails) and appropriate antibiotic therapy was started. In order to minimize the pressure in the biliary tree and prevent the bile flow through the TBF, we drained the bilioma with a 10Fr pigtail catheter and we introduced a percutaneous transhepatic biliary drainage (10Fr pigtail) with a percutaneous transhepatic approach. Regarding the BBF and BPF, we decided to treat them with percutaneous synthetic glue embolization (Glubran mixed with Lipiodol 1 : 3). Sixteen days after, there was no evidence of BBF and BPF at percutaneous cholangiography (Figure 5(a)). Consequently, we decided to remove both chest and abdomen drainages. Presently, the patient is alive and free from cancer recurrence 14 months after the last liver metastasis MWA and almost 5 years after the diagnosis of CRC (Figures 5(b) and 5(c)).

Written informed consent was obtained from the patient. All procedures were in accordance with the ethical standards of the institutional and national research committees and with the Helsinki Declaration, as revised in 2013.

## 3. Discussion and Literature Review

For patients not eligible for surgery, MWA can be offered as a valid alternative option for liver metastasis from CRC [1–3]. Despite its safe profile, MWA may have several complications including vascular injury, biliary damage, and infections [4]. The incidence of biliary tract thermal ablation complications (e.g., biliary strictures, bilomas, and bile leaks) ranges between 0.1% and 12% of cases, and TBF are exceedingly rare [5, 7, 9]. Postablation tissue inflammation can be considered the main trigger process causing adhesion between the liver and diaphragm. Furthermore, the biliary stricture and the intrapleural pressure lower than abdominal pressure play a role in the fistula formation and favor the flow of bile toward the pleural space. Consequently, the risk of this complication is higher when treating voluminous lesions located near the diaphragm (i.e., lesions in hepatic segments VII or VIII) [10–13].

Despite the diffusion of thermal ablation for the treatment of liver metastases, the literature reports higher rates of postablative TBF in patients with primary hepatic tumor than in patients with secondary liver lesions [8, 12, 14]. We speculate that hepatocarcinoma and intrahepatic cholangiocarcinoma are often voluminous and subdiaphragmatic lesions with consequently high risk of bile leaks.

The diagnosis of TBF is based on radiologic imaging. CT scan and magnetic resonance, indeed, can show indirect signs of biliopleural communication as pleural effusion, intrahepatic bile duct dilatation, and postprocedure liver damage close to the diaphragm. Conversely, cholangiography is the procedure of choice to identify the biliary leak. The combination of pathognomonic clinical symptoms, a history of recent locoregional liver procedure, and radiological imaging, guide the diagnosis [10, 15, 16].

The optimal treatment for TBF is far to be defined [7, 16]. Although the surgical approach based on debridement and ductal repair has been long advocated [6, 12], less-invasive treatments have recently developed [16–18]. None of the patients with postablative TBF for liver metastasis described in the literature underwent surgery (Table 1) [14, 16–20]. The conservative approach is aimed to decrease the pressure in the biliary tree and to prevent the flow of bile through the TBF, enhancing its closure [7]. Biliary drainage can be performed by ERCP with endoprosthesis or stent placement or by means of the PT approach [17–20]. The synthetic glue treatment is a minimally invasive and safe technique, already used for endoscopic cure of gastrointestinal fistula and as an embolizing agent in interventional radiology and vascular neuroradiology. The successful use of Glubran® glue to seal the TBF is described in few reports [16].

## 4. Conclusions

TBF is a rare postablation complication characterized by relevant mortality and morbidity rates. The diagnosis of TBF requires a high index of suspicion. Indeed, early detection and conservative management with biliary drainage may prevent severe sequelae. The current case highlights the successful management of an iatrogenic, nonhealing TBF. We initially decided to decompress the biliary system with endoscopic stent placement. However, this method offered a short-term benefit followed by pleural effusion recurrence complicated by BBF. Percutaneous drainages, biliary decompression, and the injection of glue in both BBF and PBF resulted in prompt resolution of biloptysis and pleural effusion. Glubran® glue embolization of the TBF is effective, safe, and should be considered for the treatment of TBF before recourse to surgery.

## Figures and Tables

**Table 1:** Six cases of thoracobiliary fistula following thermal ablation of liver metastases.

Author	Primary tumor	Location (hepatic segment)	Treated lesion(s) (number)	Maximum diameter (mm)	Fistula	Onset time (days)	Treatment	Outcome	Time for resolution (weeks)
Pende	Colorectal	V–VIII	1	N/A	BPF	N/A	CD + ED	Resolution	2
Tran	Colorectal	Dome	N/A	N/A	BBF	28	ED	Resolution	8
Kim	Gastric	VII	1	35	BBF	56	PD	Resolution	12
Liberale	Renal	IV	1	65	BBF	28	CD + ED	Resolution	4
Xi	Breast	Right lobe	1	NA	BBF	14	Palliative	Dead	
Pinsker	NET small bowel	V–VI–VII	3	N/A	BBF	1	ED synthetic glue^∗^	Resolution	28

^∗^Resolutive treatment. NET: neuroendocrine tumor; BPF: biliopleural fistula; BBF: biliobronchial fistula; ED: endoscopic drainage; PD: percutaneous drainage; CD: chest drainage; N/A: not acquired.

## Data Availability

The data used to support the findings of this study are available from the corresponding author upon request.
